# An Insight Into the Potentiation Effect of Potassium Iodide on aPDT Efficacy

**DOI:** 10.3389/fmicb.2018.02665

**Published:** 2018-11-19

**Authors:** Cátia Vieira, Ana T. P. C. Gomes, Mariana Q. Mesquita, Nuno M. M. Moura, M. Graça P. M. S. Neves, M. Amparo F. Faustino, Adelaide Almeida

**Affiliations:** ^1^Department of Biology and CESAM, University of Aveiro, Aveiro, Portugal; ^2^Department of Chemistry and QOPNA, University of Aveiro, Aveiro, Portugal

**Keywords:** antimicrobial photodynamic therapy, cationic porphyrins, phenothiazines, xanthenes, potassium iodide, bioluminescent *E. coli*

## Abstract

Antimicrobial photodynamic therapy (aPDT) is gaining a special importance as an effective approach against multidrug-resistant strains responsible of fatal infections. The addition of potassium iodide (KI), a non-toxic salt, is recognized to increase the aPDT efficiency of some photosensitizers (PSs) on a broad-spectrum of microorganisms. As the reported cases only refer positive aPDT potentiation results, in this work we selected a broad range of porphyrinic and non-porphyrinic PSs in order to gain a more comprehensive knowledge about this aPDT potentiation by KI. For this evaluation were selected a series of *meso*-tetraarylporphyrins positively charged at meso positions or at β-pyrrolic positions and the non-porphyrinic dyes Methylene blue, Rose Bengal, Toluidine Blue O, Malachite Green and Crystal Violet; the assays were performed using a bioluminescent *E. coli* strain as a model. The results indicate that KI has also the ability to potentiate the aPDT process mediated by some of the cationic PSs [**Tri-Py(+)-Me**, **Tetra-Py(+)-Me**, **Form**, **RB**, **MB**, **Mono-Py(+)-Me**, **β-ImiPhTPP**, **β-ImiPyTPP**, and **β-BrImiPyTPP**] allowing a drastic reduction of the treatment time as well as of the PS concentration. However, the efficacy of some porphyrinic and non-porphyrinic PSs [**Di-Py(+)-Me *opp***, **Di-Py(+)-Me *adj***, **Tetra-Py**, **TBO**, **CV**, and **MG**] was not improved by the presence of the coadjuvant. For the PSs tested in this study, the ones capable to decompose the peroxyiodide into iodine (easily detectable by spectroscopy or by the visual appearance of a blue color in the presence of amylose) were the most promising ones to be used in combination with KI. Although these studies confirmed that the generation of ^1^O_2_ is an important fact in this process, the PS structure (charge number and charge position), aggregation behavior and affinity for the cell membrane are also important features to be taken in account.

## Introduction

Antibiotics are among the most commonly prescribed drugs used in both human medicine and in farm animals, resulting in the selection of multiple drugs resistant (MDR) bacteria (Economou and Gousia, 2015; O’Neill, 2016). Infections with resistant bacteria are difficult to treat, causing severe illness and requiring costly and sometimes toxic alternatives, such as antibiotics of last resort. Drugs of last resort, such as vancomycin against Gram-positive bacteria and colistin against Gram-negative bacteria, have been the most reliable therapeutic agents against MDR bacteria. However, bacterial strains resistant to these antibiotics have been isolated worldwide (Levine, 2006; Wang et al., 2018). This resistance can result from a chromosomal gene mutation, but comes mainly from horizontal transfer from external gene sources (Chambers and DeLeo, 2009; DeLeo et al., 2010; Gardete and Tomasz, 2014; Gao et al., 2016). The development of novel antibiotics is not likely to solve the problem and it is probably only a matter of time until they will be also ineffective. Bacteria will inevitably find ways of resisting to the conventional antibiotics, which is why alternative approaches areurgent.

**FIGURE 1:**
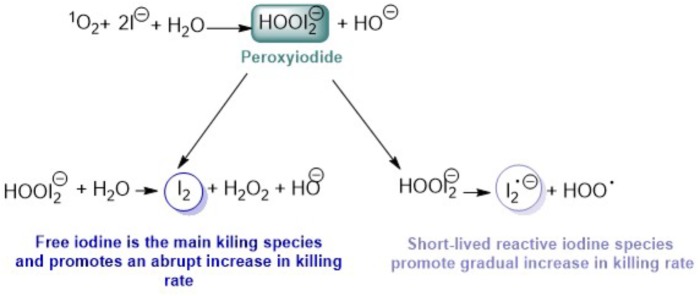
Schematic representation of the decomposition of peroxyiodide produced by the reaction of ^1^O_2_ and KI (elaborated according with the literature – Vecchio et al., 2015; Zhang et al., 2015; Freire et al., 2016; Gsponer et al., 2016; Hamblin, 2017; Huang et al., 2017, 2018a; Kashef et al., 2017; Reynoso et al., 2017; Wen et al., 2017).

Antimicrobial photodynamic therapy (aPDT) can be a very promising alternative to antibiotic treatment namely in localized infections (Dai et al., 2010). aPDT involves the use of a photosensitizer (PS) which in the presence of visible light and oxygen produces reactive oxygen species (ROS), such as singlet oxygen (^1^O_2_). These species are responsible for the oxidation of several cellular components conducting to rapid cell inactivation. This approach presents some advantages when compared with the use of antibiotics, such as being efficient independently of the microorganism antibiotic resistance profile (Jori et al., 2011), does not induce the development of resistance, even after several cycles of treatment (Giuliani et al., 2010; Tavares et al., 2010; Costa et al., 2011) and can be applied with efficacy against Gram-negative and Gram-positive bacteria. aPDT is considered more effective against Gram-positive bacteria due to their highly permeable cell walls allowing the easy diffusion of neutral, positive and negative charged PS into the cell. However, the impermeable external membrane of Gram-negative bacteria cell wall limits the anionic or neutral-charge PSs entrance (Minnock et al., 2000). This limitation is overcome by the use of cationic PS. These PSs are able to bind and penetrate into the cell wall by the “self-promoted uptake pathway” (Hancock et al., 1991; Merchat et al., 1996). Nevertheless, neutral PSs or PSs with low number of charges can be effective against this type of bacteria by coupling or combining them with positively charged entities such as poly-L-lysine, polyethylenimine and polymyxin B nonapeptide that act as membrane disruptors (Nitzan et al., 1992; Helander et al., 1997; Lounatmaa et al., 1998; Soukos et al., 1998). Ethylenediaminetetraacetic acid (EDTA) is also commonly used to destabilize the native organization of Gram-negative wall (Yoshimura and Nikaido, 1985; Jori et al., 2006). It has also been shown that different organic salts can improve the efficiency of aPDT against Gram-negative bacteria (Huang et al., 2012; Kasimova et al., 2014). Recently, some studies have demonstrated that aPDT can be potentiated by addition of several different inorganic salts, such as sodium bromide (Wu et al., 2016) sodium azide (Huang et al., 2012; Kasimova et al., 2014), sodium thiocyanate (St Denis et al., 2013) and potassium iodide (Vecchio et al., 2015; Zhang et al., 2015; Freire et al., 2016; Huang et al., 2016, 2017, 2018a,c; Hamblin, 2017; Reynoso et al., 2017; Wen et al., 2017). In fact, the addition of iodide has been shown to improve the efficiency of aPDT in several animal models of localized infection. This salt is non-toxic and is an approved drug for antifungal therapy (Hamblin, 2017). The studies involving the use of KI demonstrate that the combination of this salt with neutral porphyrins, fullerenes and other dyes gives rise to higher microbial inactivation rates when are compared to the use of the PSs alone. KI was firstly studied as potentiator of aPDT mediated by a C_60_ fullerene bisadduct (Zhang et al., 2015). The results showed that KI potentiated the ultraviolet A (UVA) or the white light-mediated killing of Gram-negative bacteria *Acinetobacter baumannii*, Gram-positive methicillin-resistant *Staphylococcus aureu*s and fungal yeast *Candida albicans*, increasing the effect in 1–2 logs. This extra killing effect was also observed *in vitro* and *in vivo* using a mouse model with an infected skin abrasion (Zhang et al., 2015). These promising results conducted to new studies concerning the mechanism of action involved. The KI effect using Methylene Blue (MB) as PS in the photoinactivation of *Escherichia coli* and *S. aureus* was also evaluated (Vecchio et al., 2015). The results showed that the addition of KI increased the bacterial killing in 4 and 2 logs for *S. aureus* and *E. coli*, respectively, in a dose-dependent manner. The authors also affirmed that the KI potentiator effect in these aPDT studies mediated by MB was probably due to the formation of reactive iodine species that were quickly produced with a short lifetime (Vecchio et al., 2015). Since then, some other examples of the potentiation of aPDT effect using combinations of PSs and KI were reported. For instance, MB and new methylene blue (NMB) were studied in the photoinactivation of oral *C. albicans* infection in a mouse model (Freire et al., 2016), Photofrin in the photoinactivation of several Gram-negative bacteria (Huang et al., 2017), BODIPY dyes in the photoinactivation of *S. aureus*, *E. coli*, and *C. albicans* (Reynoso et al., 2017). This approach was also efficient in aPDT of Gram-negative and Gram-positive bacteria mediated by Rose Bengal (Wen et al., 2017) and fullerenes (Huang et al., 2018b). Interestingly, an anionic porphyrin in the presence of KI was able to photoinactivate *E. coli* (Huang et al., 2018a). The combination of MB and KI was also efficient to treat an urinary tract infection in a female rat model (Huang et al., 2018c). All these reports helped to elucidate the mechanism of action of KI potentiation. It was proposed that the extra killing effect is caused by several parallel reactions initiated by the reaction of ^1^O_2_ with KI producing peroxyiodide (Figure 1), that can suffer further decomposition by two different pathways, which are dependent on the degree of binding of the PS to the microbial cells (Vecchio et al., 2015; Zhang et al., 2015; Freire et al., 2016; Gsponer et al., 2016; Hamblin, 2017; Huang et al., 2017, 2018a; Kashef et al., 2017; Reynoso et al., 2017; Wen et al., 2017). One of the pathways involves the formation of free iodine (I_2_/${\text{I}}_{3}^{-}$) and hydrogen peroxide (H_2_O_2_). Free iodine can kill microbial cells when generated in solution but needs to reach a sufficient threshold concentration to be microbicidal. The amount of free iodine produced depends on the amount of ^1^O_2_ produced, but also on the concentration of iodide anion present in solution (Figure 1). The other one involves a homolytic cleavage process producing reactive iodine radicals (${\text{I}}_{2}^{\cdot -}$), which are much more toxic if generated very close to the target cells since these radicals have short diffusion distance (Figure 1).

The microbial killer role of the two species can be distinguished by observing the killing microbial curve profile. When the principal contribution for the killing is the free iodine, the curves assumes an abrupt threshold value. On the other hand, a gradual killing curve can be observed when the short-lived reactive iodine species are the mainly killing species (Huang et al., 2018a).

**FIGURE 2:**
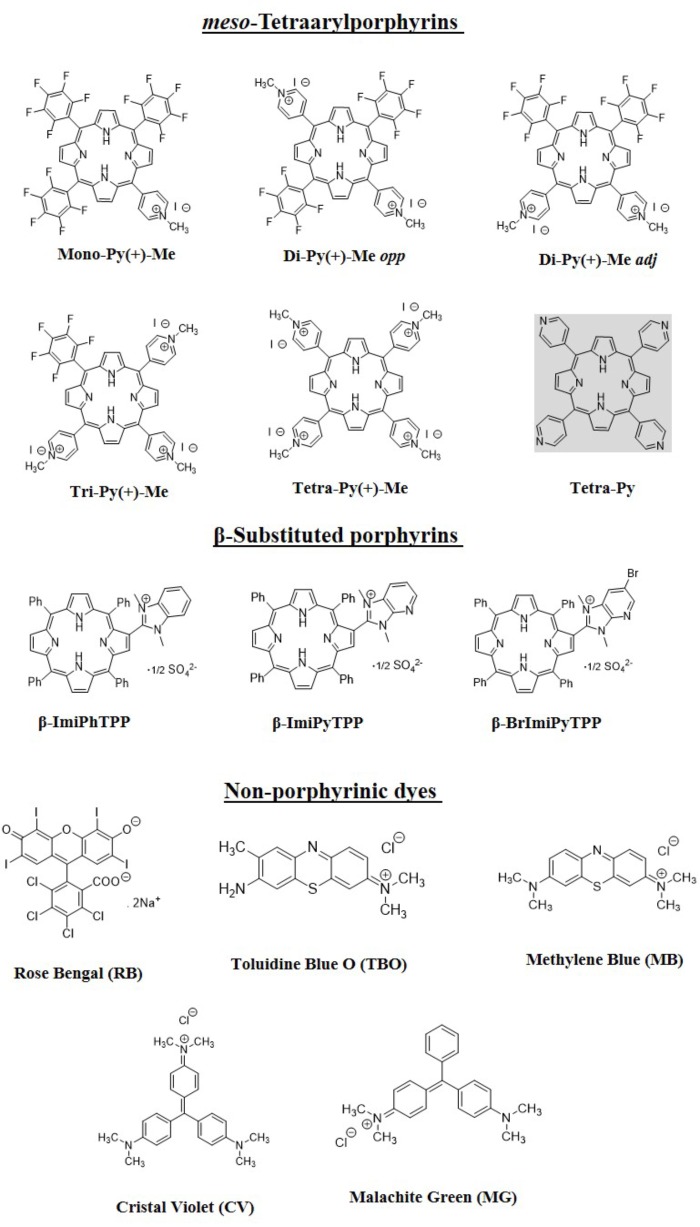
Structures and acronyms/abbreviations of the PSs used in this study.

Until now, the literature survey only reported combinations of PSs and KI with a positive aPDT potentiation. Additionally, the possibility of extending the approach to cationic porphyrins was not evaluated. Consequently, in this work, in order to gain a more comprehensive knowledge about this type of potentiation, we decided to assess the effect of KI in the presence of a broad range of cationic porphyrinic and non-porphyrinic dyes as PSs (Figure 2). To achieve this objective and considering the high number of assays required to evaluate the different combinations of PSs with KI, the assays were performed using a bioluminescent *E. coli* strain as a bacterial model. It is well known that the bioluminescence approach can provide a sensitive and innocuous way to detect the viability state of microorganisms. Compared to the conventional plating count methodology, the use of bioluminescent strains in aPDT allows to monitor the process in real-time and it is a sensitive and cost-effective methodology to evaluate this effect. Moreover, the strong correlation between CFU and bioluminescent signal of the bioluminescent *E. coli* used in this work has already been proved and described (Alves et al., 2008, 2011a,b).

The structures of the selected PSs summarized in Figure 2 comprise: (i) the five structurally related *meso*-tetraarylporphyrins with one [**Mono-Py(+)-Me**], two [**Di-Py(+)-Me *opp*** and **Di-Py(+)-Me *adj***], three [**Tri-Py(+)-Me**], and four [**Tetra-Py(+)-Me**] positives charges and a formulation (**Form**) based on these porphyrins; (ii) the three β-substituted porphyrins **β-ImiPhTPP**, **β-ImiPyTPP**, and **β-BrImiPyTPP** bearing positively charged imidazole units; and (iii) the non-porphyrinic dyes – methylene blue (**MB**), Rose Bengal (**RB**) and Toluidine Blue O (**TBO**), crystal violet (**CV**) and malachite green (**MG**).

In the selection of these three series of PSs was considered their different photoinactivation profile toward *E. coli* and their mechanism of action (Type I and Type II).

For the *meso*-tetraarylporphyrins with positive charges at the *meso* position the studies already performed demonstrated that their photodynamic efficiency was dependent on charge number, charge distribution, aggregation behavior and molecular amphiphilicity and the order of their efficacy was: **Mono-Py(+)-Me** < **Di-Py(+)-Me *opp*** < **Di-Py(+)-Me *adj*** < **Tetra-Py(+)-Me** < **Tri-Py(+)-Me**. Additionally, a formulation (**Form**) constituted by a non-separated mixture of Mono-Py(+)-Me (19%), Di-Py(+)-Me *opp* and Di-Py(+)-Me *adj* (20%) Tri-Py(+)-Me (44%) and Tetra-Py(+)-Me (17%) was also studied. This mixture has already proved to be efficient in the photoinactivation of *S. aureus*, *E. coli* and *Pseudomonas syringae* pv. *actinidiae* and is considered an excellent alternative to the highly efficient **Tri-Py(+)-Me** since the production costs and also the production time was reduced significantly (Marciel et al., 2018; Martins et al., 2018). The neutral 5,10,15,20-tetra-(4-pyridyl)porphyrin (**Tetra-Py**) precursor of the positively charged **Tetra-Py(+)-Me** was also included.

For the *meso*-tetraarylporphyrins with a positive charge at the *beta*-pyrrolic position (**β-ImiPhTPP**, **β-ImiPyTPP**, and **β-BrImiPyTPP**) a different efficacy profile in photoinactivation of *E. coli* at concentrations of 20 μM was observed in previous studies; however, at 5.0 μM none of the three PSs caused a significant decrease in bacterial activity (Moura et al., 2019).

Although porphyrins and porphyrins analogs comprise most of the PSs used in aPDT, several non-porphyrinic chromogens exhibit photodynamic activity (Ormond and Freeman, 2013). Thus, for this study were selected good ^1^O_2_ generators with positive charges that already proved their photodynamic efficiency in clinical trials such as the phenothiazinium salts **MB** and **TBO** (Abrahamse and Hamblin, 2016). In this study were also included two photoactive dyes that act mainly through type I mechanism (with lower ^1^O_2_ production rates), the **CV** and **MG**. In this evaluation the study was extended to the xanthene derivative **RB**. Combinations of KI with **RB** and with **MB** were already studied and were introduced in this work to corroborate our results (Vecchio et al., 2015; Wen et al., 2017).

## Materials and Methods

### Photosensitizers: Stock Solutions and UV-Vis Spectra

Stock solutions of each porphyrin were prepared at 500 μM in dimethyl sulfoxide (DMSO) and stored in the dark. Stock solutions of non-porphyrinic dyes were prepared at 500 μM in phosphate buffer solution (PBS) and stored in the dark.

The porphyrins 5-(1-methylpyridinium-4-yl)-10,15,20-tris(pentafluorophenyl)-porphyrin mono-iodide [**Mono-Py(+)-Me**], 5,15-bis(1-methylpyridinium-4-yl)-10,20-bis(pentafluorophenyl)porphyrin di-iodide [**Di-Py(+)-Me *opp***] 5,10-bis(1-methylpyridinium-4-yl)-15,20-bis(pentafluorophenyl)-porphyrin di-iodide [**Di-Py(+)-Me *adj***], 5,10,15-tris(1-methylpyridinium-4-yl)-20-(pentafluorophenyl)porphyrin tri-iodide [**Tri-Py(+)-Me**] and 5,10,15,20-tetrakis(1-methylpyridinium-4-yl)porphyrin tetra-iodide [**Tetra-Py(+)-Me**], the formulation (**Form**) of the non-separated porphyrins Mono-Py(+)-Me (19%), Di-Py(+)-Me *opp* and Di-Py(+)-Me *adj* (20%) Tri-Py(+)-Me (44%) and Tetra-Py(+)-Me (17%) and the neutral 5,10,15,20-tetra-(4-pyridil)porphyrin (**Tetra-Py**) were synthetized according with the literature (Simões et al., 2016; Marciel et al., 2018; Martins et al., 2018). The preparation of the mono-cationic porphyrins **β-ImiPhTPP**, **β-ImiPyTPP**, and **β-BrImiPyTPP** bearing an imidazole ring at the β-pyrrolic position were synthetized according with a procedure developed in our laboratory (Moura et al., 2019), Crystal Violet (**CV**) was purchased from Merck, Rose Bengal (**RB**) from Fluka AG, Malachite Green (**MG**) from Riedel-de-Haën^TM^, Methylene Blue (**MB**) and Toluidine Blue O (**TBO**) from Acros Organics. The UV-Vis spectra of the PSs are presented in Supplementary Figure S2 (see Supporting Information).

### Light Sources

The potentiation of aPDT effect between the PS and KI was evaluated by exposing the bacterial suspension in the presence of each combination to a set of fluorescent PAR lamps which is constituted by 13 fluorescent lamps OSRAM 21 of 18 W each, PAR white radiation (380–700 nm) at an irradiance of 25 W m^−2^. All the irradiances were measured with a Power Meter Coherent FieldMaxII-Top combined with a Coherent PowerSens PS19Q energy sensor.

### Bacterial Strains and Growth Conditions

The genetically transformed bioluminescent *E. coli* Top10 (Alves et al., 2011b) was grown on Tryptic Soy Agar (TSA, Merck) supplemented with 50 mg mL^−1^ of ampicillin (Amp) and with 34 mg mL^−1^ of chloramphenicol (Cm). Before each assay, one isolated colony was transferred to 10 mL of tryptic soy broth medium (TSB, Merck) previously supplemented with Amp and Cm and was grown overnight at 25°C under stirring (120 rpm). An aliquot was transferred into 10 mL TSB under the same growth conditions till stationary growth phase was achieved. An optical density at 600 nm (OD_600_) of 1.6 ± 0.1 corresponded to ≈10^8^ colony forming units (CFU) mL^−1^.

The correlation between CFU mL^−1^ and the bioluminescent signal (in RLUs) of bioluminescent *E. coli* strain was evaluated. A fresh overnight bacterial culture was serially diluted (10^−1^ to 10^−9^) in PBS. Non-diluted and diluted aliquots were pour-plated on TSA medium (0.5 mL) and, simultaneously, were read on a luminometer (0.5 mL) (TD-20/20 Luminometer, Turner Designs, Inc., Madison, WI, United States) to determine the bioluminescence signal. The results obtained are presented in Supplementary Figure S1 (see Supporting Information).

### Antimicrobial Photodynamic Therapy (aPDT) Procedure

Bioluminescent *E. coli* culture was grown overnight and was tenfold diluted in PBS (pH 7.49), to a final concentration of ∼10^8^ CFU mL^−1^, which corresponds approximately to 10^8^ RLU. The bacterial suspension was equally distributed in 50 mL sterilized and acid-washed beakers.

#### Bioluminescence Monitoring

All the experiments were carried out under PAR white light (380–700 nm) and the *E. coli* bioluminescence signal was measured in the luminometer at different times of light exposure. The assays were finished whenever the detection limit of the luminometer was achieved (*c.a* 2.3 log). Light control (LC), dark control (DC), and KI control, were also evaluated as described below.

#### Evaluation of the Inorganic Salt Effect on Tetra-Py(+)-Me Photodynamic Action

The first experiments were performed in order to assess the effect of different inorganic salts in the inactivation of *E. coli* through aPDT approach using the tetracationic porphyrin **Tetra-Py(+)-Me,** extensively studied in bacterial photoinactivation processes (Alves et al., 2008; Tavares et al., 2011; Simões et al., 2016). The selected inorganic salts were KI, NaI, KCl, NaCl, and NaBr and the assays were conducted with 50 mM of each salt and 5.0 μM of **Tetra-Py(+)-Me**. All the inorganic salts were purchased from Sigma-Aldrich (St. Louis, MO, United States) and stock solutions were prepared at 500 mM in PBS immediately before each experiment.

The assays were carried out by exposing the bioluminescent *E. coli* suspension to **Tetra-Py(+)-Me** at 5.0 μM with each salt added from the stock solution to achieve the final concentrations of 50 mM. Simultaneously, the following different controls were performed: one light control (LC) that contained a bacterial suspension exposed to the same light conditions as the samples, and dark controls (DC) that comprised a bacterial suspension incubated with the PS at 5.0 μM and with the distinct salts at 50 mM. DC were protected from light during all the procedure. The samples and controls were protected from light with aluminum foil and remained in the dark for 15 min to promote the porphyrin binding to *E. coli* cells before irradiation. Then, both samples and controls were exposed to the PAR white light at 25 W m^−2^ under stirring (120 rpm) and placed on a tray; the beaker bottoms were covered with water to maintain the samples at constant temperature (25°C). Finally, aliquots of 0.8 mL of samples and controls were collected at different times of light exposure and the bioluminescence signal was measured in the luminometer. Three independent experiments with two replicates were performed and the results were averaged.

#### Evaluation of the Antimicrobial Effect in the Presence of Different PSs and KI

The assays were carried out by exposing a final volume of 10 mL of a bioluminescent *E. coli* suspension to each PS at 5.0 μM and combinations of each PS at 5.0 μM and KI concentrations at 50 and 100 mM and for **RB**, **CV**, **MG** also at 25 mM. The samples were protected from light with aluminum foil and incubated in the dark for 15 min. Light and dark controls were also carried out simultaneously with the aPDT procedure: the light controls (LC) comprised a bacterial suspension and a bacteria suspension with KI at 100 mM exposed to the same light protocol; and the dark control (DC) comprised a bacterial suspension incubated with the PSs at 5.0 μM and KI at the higher concentration tested (100 mM) protected from light. The aPDT treatment was performed as described above. Three independent experiments with two replicates were performed and the results were averaged.

#### Detection of Iodine Formation

In a 96 wells microplate, appropriate volumes of each PS at 5.0 μM (1 μL) and combinations of each PS at 5 μM (1 μL) and KI at 100 mM (2 μL) were added to each well and irradiated with PAR white light at 25 W m^−2^. The generation of iodine was monitored by reading the absorbance at 340 nm at irradiation times 0, 5, 10, 15, 30, 45, 60, 75, 90, 105, and 120 min. As positive control it was used Lugol’s solution diluted to 1:1000.

Another simple assay to detect iodine was also performed, for the different combinations of PS and KI, in the presence of amylose due to the well-known formation of a strong blue complex when these two species are present (Luallen, 2017). So, to the beakers containing a starch solution at a concentration of 2 mg L^−1^, it was added each PS at 5 μM and KI at 100 mM. The samples were incubated in the dark for 15 min and afterward were exposed continuously and under stirring (120 rpm) to the same light source used in the aPDT assays. The color change was registered and photographed at different times of irradiation for each sample. At the same time, the following control assays were performed: PS + light; KI + light, PS + KI under dark.

### Statistical Analysis

Three independent experiments with two replicates per assay for each condition were done. The statistical analysis was performed with GraphPad Prism. Normal distributions were checked by the Kolmogorov–Smirnov test and the homogeneity of variance was verified with the Brown Forsythe test. ANOVA and Dunnet’s multiple comparison tests were applied to assess the significance of the differences between the tested conditions. A value of *p* < 0.05 was considered significant.

## Results

The effect of KI for each series of PSs toward *E. coli* was evaluated using the same concentration of PS (5.0 μM) and KI concentrations of 50 and 100 mM (unless other concentrations were mentioned) under PAR white light at an irradiance of 25 W m^−2^. These KI concentrations were selected considering the ones referred in similar studies and knowing that higher concentrations can limit the combined protocol application in clinic area due to osmotic stress. The PS, **TetraPy(+)-Me,** was selected to confirm the benefic effect of KI among other inorganic salts (NaI, NaCl, KCl, and NaBr). This well-known tetracationic porphyrin is extensively studied in bacterial photoinactivation processes and is considered an excellent reference when the efficacy of different cationic porphyrins are compared (Alves et al., 2008; Tavares et al., 2011; Simões et al., 2016). Low light doses ranging from 1.5 to 36 J/cm^2^ emitted by a fluorescent lamp set (380–700 nm) were selected based on their efficacy to inactivate a large range of microorganisms (Marciel et al., 2017; Moura et al., 2019). Additionally, this light source was able to accomplish the required overlap between PS absorption and light setup emission spectrum (see Supplementary Figure S2; Costa et al., 2010; Cieplik et al., 2015).

### Evaluation of the Salt Effect on Tetra-Py(+)-Me Photodynamic Efficiency

**FIGURE 3:**
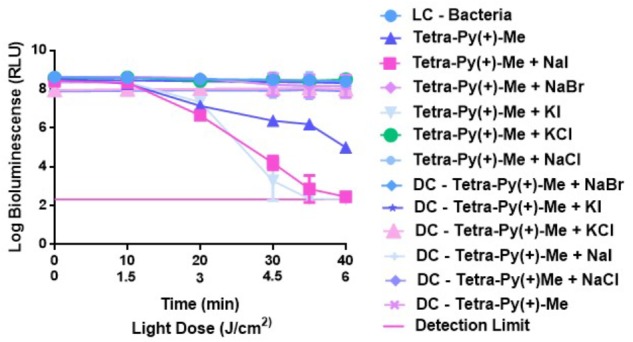
Survival of bioluminescent *E. coli* during aPDT with **Tetra-Py-(+)-Me** at 5.0 μM and 50 mM of KI, NaI, KCl, NaCl, and NaBr after irradiation with PAR white light (380–700 nm) at an irradiance of 25 W m^−2^ for 40 min. The values are expressed as the three independent experiments; error bars indicate the SD and in some cases are collapsed with the symbols.

The results presented in Figure 3 show that the photoinactivation pattern of *E. coli* in the presence of **Tetra-Py(+)-Me** is strongly dependent on the anion used.

The results clearly indicate that when combinations of **Tetra-Py(+)-Me** with KI and NaI were used, a reduction of the bioluminescence signal of *c.a.* 4 log was observed after 30 min of irradiation. In the case of NaBr, KCl and NaCl no potentiation on the aPDT effect was detected. Light and dark controls showed no significant variation in the bioluminescence produced by *E. coli*.

### Evaluation of the KI Effect on the Photodynamic Action of *Meso*-Tetraarylporphyrins Bearing One to Four Positive Charges

**FIGURE 4:**
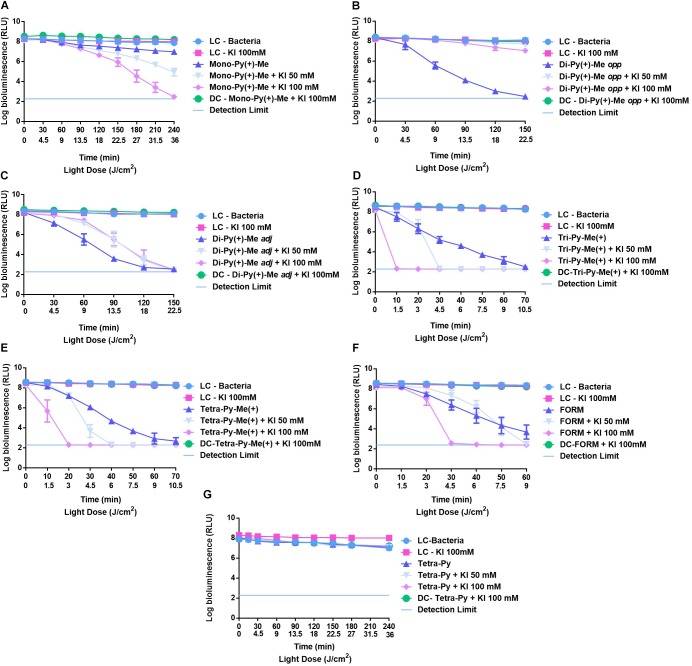
Survival of bioluminescent *E. coli* during aPDT assays in the presence of **Mono-Py(+)-Me** **(A)**, **Di-Py(+)-Me *opp*** **(B)**, **Di-Py(+)-Me *adj*** **(C)**, **Tetra-Py(+)-Me** **(D)**, **Tri-Py(+)-Me** **(E)**, **Form** **(F)**, and **Tetra-Py** **(G)** at 5.0 μM alone and combined with KI at 50 and 100 mM. The values are expressed as the three independent experiments; error bars indicate the SD.

The effects of KI at 50 and 100 mM in the photodynamic action of **Mono-Py(+)-Me**, **Di-Py(+)-Me *opp***, **Di-Py(+)-Me *adj***, **Tri-Py(+)-Me**, and **Tetra-Py(+)-Me** toward *E. coli* are summarized in Figure 4.

In the cases of the LCs (Bacteria and bacteria + KI irradiated) and DC (bacteria + PS + KI in the dark) no decrease in *E. coli* bioluminescent signal was detected. These results indicate that the viability of this recombinant bioluminescent bacterium was not affected by irradiation, by the presence of the salt or by any of the tested combinations of PS + KI in the dark.

The results shown in Figure 4A for the monocationic porphyrin [**Mono-Py(+)-Me**] demonstrated that its low efficacy is strongly improved by the presence of KI; the poor activity of this PS toward *E. coli* was previously related with its low water solubility leading to aggregation and, consequently, to low ^1^O_2_ generation. Under the conditions used in these assays this porphyrin maintained its low efficacy causing a decrease on *E. coli* bioluminescence signal of 0.9 log (*p* < 0.0001) after 240 min of irradiation. However, the addition of KI at 50 mM and 100 mM potentiated the effect of this mono-cationic porphyrin, causing bioluminescent signal reductions of *c.a.* 3.5 and 5.5 log (*p* < 0.0001) after 150 min of irradiation.

The dicationic porphyrins **Di-Py(+)-Me *opp*** and **Di-Py(+)-Me *adj*** without the presence of the coadjuvant promoted similar effects on the reduction (*c.a.* 6 log after, respectively, 150 and 120 min of irradiation) of *E. coli* bioluminescence signal (Figures 4B,C). However, when these two isomers were combined with KI the results obtained were significantly different. The combination of **Di-Py(+)-Me *adj*** with KI at 50 and 100 mM produced similar results in the photoinactivation of bioluminescent *E. coli* and no improvement in aPDT efficiency was detected (Figure 4C). In fact, in the last irradiation time, there were no significant differences in the *E. coli* bioluminescence signal promoted by **Di-Py(+)-Me *adj*** and the two combinations of **Di-Py(+)-Me *adj*** + KI. In the case of **Di-Py(+)-Me opp** (Figure 4B) the presence of KI (at 50 and 100 mM) led to a significant reduction on its efficacy. The maximum inactivation achieved for the combination of this PS with 100 mM of KI was 1.7 log (*p* < 0.0001).

The **Tetra-Py(+)-Me** and **Tri-Py(+)-Me** were the most efficient porphyrins in the photoinactivation of bioluminescent *E. coli*, which is also in accordance with the literature (Simões et al., 2016). These porphyrins, when acting by themselves, showed to be potent PSs for the inactivation of bioluminescent *E. coli*, demanding short irradiation times (*c.a.* 70 min) to achieve total photoinactivation of this Gram-negative bacterium (Figures 4D,E). The combination of these PSs with KI at 50 and 100 mM increased dramatically the effect of these PSs in the photoinactivation of bioluminescent *E. coli* (Figures 4D,E). In the case of **Tri-Py(+)-Me**, it was observed an abrupt decrease in *E. coli* viability after 30 and 10 min of irradiation when the combinations of this PS with 50 mM and 100 mM of KI were used, respectively (Figure 4D). This sharp decrease was also observed for the combination of **Tetra-Py(+)-Me** and KI; after 30 and 10 min of irradiation no bioluminescent signal was detected for combinations **Tetra-Py(+)-Me** +KI 50 mM and **Tetra-Py(+)-Me** +KI 100 mM, respectively.

These results prompted us to study the effect of KI in the aPDT efficiency of the porphyrinic formulation (**Form**) described as an excellent alternative to the highly efficient **Tri-Py(+)-Me,** as it was mentioned above. The results summarized in Figure 4F show that this formulation at 5 μM in the absence of the coadjuvant and after 60 min of irradiation, promoted a decrease in the bioluminescence signal of *E. coli* of 4 log (*p* < 0.0001) (Figure 4F). When the assays were repeated in the presence of KI at 50 mM a more pronounced decrease in *E. coli* viability was detected after 40 min of irradiation, reaching the detection limit of the luminometer after 60 min. This rapid decrease in the viability of this bacterium occurred even sooner, after only 20 min of irradiation, when KI was used at 100 mM.

In order to check if the presence of positive charges is a required feature for the combination of KI with this series of porphyrins, the efficacy of the neutral 5,10,15,20-tetra(4-pyridyl)porphyrin (**Tetra-Py**) was evaluated in the presence of this salt at 50 and 100 mM. In Figure 4G are summarized the results obtained and it was verified that the low efficacy of this neutral porphyrin was not improved by the presence of the salt, suggesting that when an increment effect was observed in the presence of KI in this series of porphyrins, the presence of at least one positive charge is mandatory.

### Evaluation of the KI Effect on the Photodynamic Action of Porphyrin Derivatives Bearing Cationic Imidazole Units at the β-Pyrrolic Position

**FIGURE 5:**
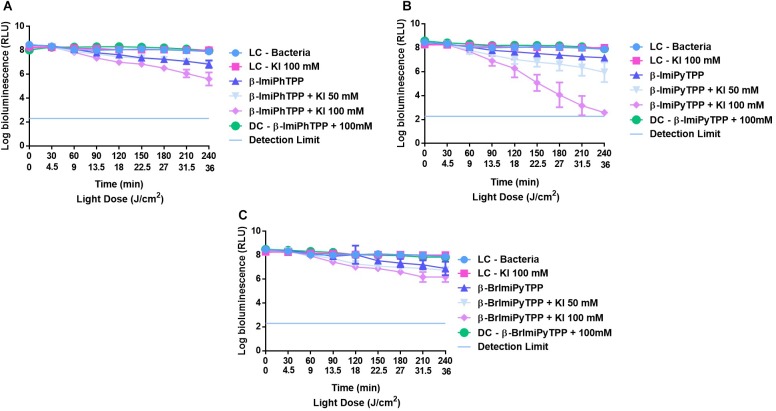
Survival of bioluminescent *E. coli* during aPDT assays in the presence of mono-cationic porphyrins **β-ImiPhTPP (A)**, **β-ImiPyTPP (B)**, and **β-BrImiPyTPP (C)** at 5.0 μM alone or combined with KI at 50 and 100 mM. The values are expressed as the three independent experiments; error bars indicate the SD.

The results obtained in the photoinactivation of bioluminescent *E. coli* with the monocationic porphyrins **β-ImiPhTPP**, **β-ImiPyTPP**, and **β-BrImiPyTPP** bearing an imidazole moiety at the β-pyrrolic position, both in the absence and in the presence of KI are presented in Figure 5. The low activity of these porphyrins at 5.0 μM in the photoinactivation of bioluminescent *E. coli* was improved in the presence of KI, although the inactivation increment was different. The combination of **β-BrImiPyTPP** and **β-BrImiPhTPP** with KI at 100 mM promoted a significant positive effect in the photoinactivation of *E. coli* with an increment on the bioluminescent reduction of 1.3 and 1.1 log for **β-ImiPhTPP** and **β-BrImiPyTPP** (*p* < 0.0001), respectively, after 240 min of irradiation when compared with the effect of these PSs in the absence of KI (Figures 5A,C).

A different profile was observed for porphyrin derivative **β-ImiPyTPP**. The best results were obtained with the combination of this PS with 100 mM of KI, promoting a significant decrease in *E. coli* viability (Figure 5B). The bioluminescence signal reduction reached the method detection limit after 240 min; when compared with the effect of these PS in the absence of KI an increment on the bioluminescent reduction of 5.3 log in cell viability was observed (*p* < 0.0001).

### Evaluation of the KI Effect in the Photodynamic Action of Non-porphyrinic Dyes

**FIGURE 6:**
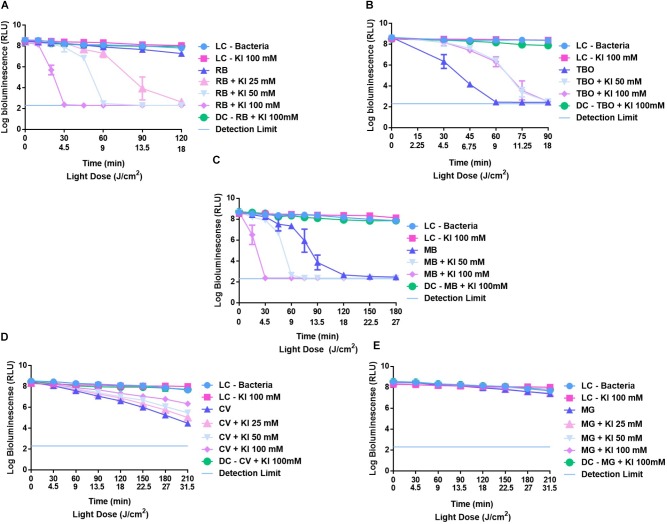
Survival of bioluminescent *E. coli* during aPDT assays in the presence of non-porphyrinic PSs at 5.0 μM **RB** **(A)**, **TBO** **(B)**, **MB** **(C)**, **CV** **(D)**, and **MG** **(E)** alone and combined with KI at 25, 50, and 100 mM. The values are expressed as the three independent experiments; error bars indicate the SD.

In Figure 6 are summarized the effects of KI at 50 and 100 mM in the photodynamic inactivation of *E. coli* when using **RB** (A), **TBO** (B), **MB** (C), **CV** (D), and **MG** (F). Combinations of **RB** (Figure 6A) and **MB** (Figure 6C) at 5.0 μM and KI showed to have a potential effect in the photodynamic inactivation of *E. coli*, causing marked reductions in the *E. coli* viability when compared with the results obtained with these dyes alone. The PS **RB**, when acting alone, promotes a decrease of 1.3 log (*p* < 0.0001) in *E. coli* viability after 150 min of irradiation. When combined with KI, an efficient decrease in bioluminescent signal of *E. coli* was observed, even when KI at 25 mM was used. At this concentration, the combination of **RB** 5.0 μM + KI 25 mM, caused a sharp decrease in the *E. coli* viability after 90 min of irradiation, reaching the detection limit of the luminometer after 120 min. This marked effect was also observed when **RB** was combined with 50 mM of KI, but it was with the combination of **RB** 5.0 μM+ KI 100 mM that this effect became more noteworthy; after 20 min of irradiation it was observed a decrease of 6 log (*p* < 0.0001) in *E. coli* viability and after 30 min no bioluminescent signal was observed.

A similar profile was observed with combinations of **MB** at 5.0 μM and KI. In the absence of KI, **MB** caused a decrease in the bioluminescence signal of *E. coli* of 5.5 log (*p* < 0.0001) after 180 min of irradiation, but when combinations of this PS with KI were used, an efficient decrease in the viability of this bacterium was also observed, after 30 and 60 min of irradiation, with KI at 100 and 50 mM, respectively.

In the cases of **TBO**, **CV**, and **MG**, a potentiation of their photodynamic action mediated by the presence of KI was not observed. In fact, **TBO** when acting alone at 5.0 μM revealed to be an excellent PS for the inactivation on bioluminescent *E. coli*, promoting a remarkable decrease in the bioluminescent signal of 6 log (*p* < 0.0001) after 60 min of irradiation. In the presence of KI, this reduction was only observed after 90 min of irradiation.

**CV** when acting alone caused a decrease in the bioluminescent signal of 3.2 log (*p* < 0.0001), however, in the presence of KI at 25, 50, and 100 mM the decrease did not go beyond 1.4, 2.2, and 2.7 log (*p* < 0.0001), respectively.

In the case of **MG** no significant effect was observed in the *E. coli* viability either when this dye was used alone or combined with KI.

### Detection of Iodine Formation Mediated by the PS

**FIGURE 7:**
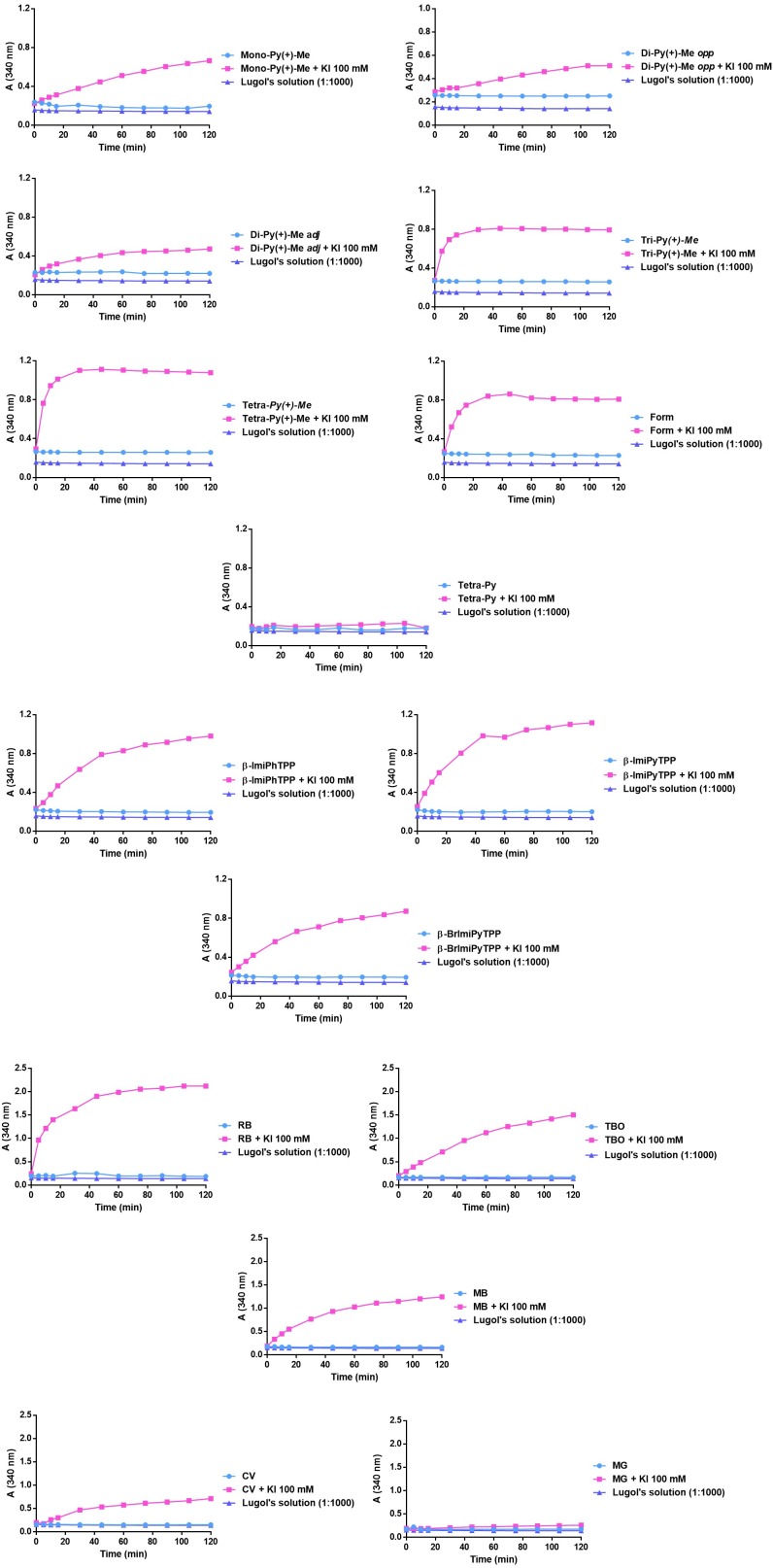
Monitoring of the formation of iodine at 340 nm after different irradiation periods in the presence of each PS at 5.0 μM and combinations of each PS at 5.0 μM and KI at 100 mM.

In order to clarify if the photodynamic improvement was related with the iodine generation from KI by the PS, the different PSs (5.0 μM) were irradiated both in the absence and in the presence of that coadjuvant at 100 mM. To verify the generation of iodine, the absorbance at 340 nm was read after 0, 5, 10, 15, 30, 45, 60, 75, 90, 105, and 120 min of irradiation. The results obtained are summarized in Figure 7.

The results had shown that the combination of KI with **Tri-Py(+)-Me**, **Tetra-Py(+)-Me**, and **Form** causes a higher production of I_2_, leading to a sharp increase in absorbance at 340 nm in the first 20 min of irradiation. On the other hand, the combination of KI with **Mono-Py(+)-Me**, **Di-Py(+)-Me *adj***, **Di-Py(+)-Me *opp*** only was able to induce a gradual increase of the absorbance at 340 nm, thus indicating the lower ability to produce I_2_. The combination of **Tetra-Py** + KI did not produce I_2_.

The gradual increase in the absorbance at 340 nm was also observed in the case of mono-cationic porphyrins **β-ImiPhTPP**, **β-ImiPyTPP**, and **β-BrImiPyTPP**. However, in the case of **β-ImiPyTPP**, the absolute value of absorbance at 340 nm after 40 min of irradiation was higher than the values observed for the other PSs, indicating the formation of higher amounts of I_2_ in this case.

In the case of the non-porphyrinic dyes, the combination of KI with **MB** and **RB** demonstrated a higher ability to produce I_2_, with a sharp increase in the absorbance at 340 nm, after 30 min of irradiation. However, combinations of **TBO**+ KI and **CV**+ KI only produced a gradual increase in the absorbance, indicating the lower capability to produce I_2_. Combination of **MG**+ KI did not promote the formation of I_2_.

**FIGURE 8:**
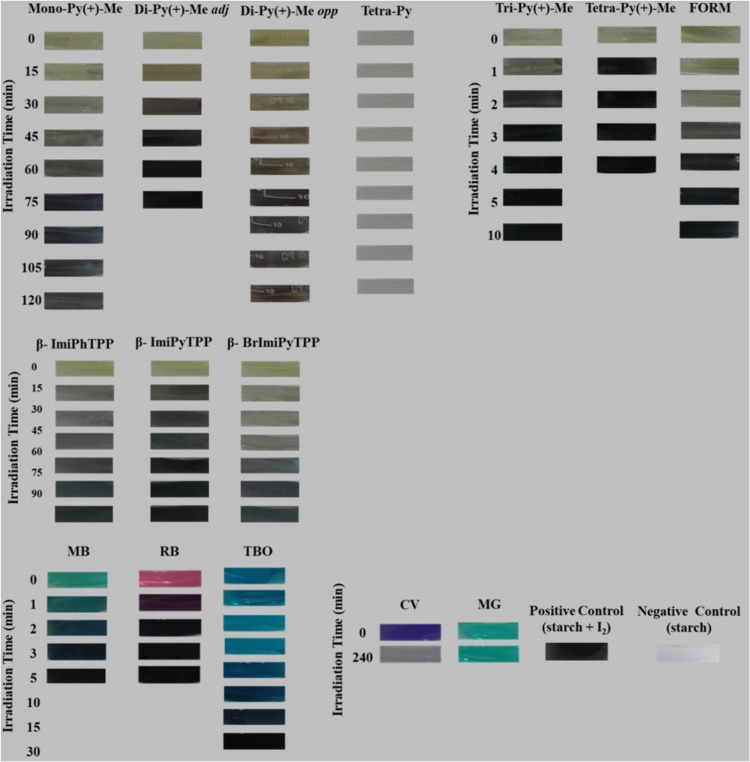
Visual appearance of the starch solutions after different irradiation periods in the presence of each PS at 5.0 μM and KI at 100 mM.

The visual appearance of the starch solutions after different irradiation periods are presented in Figure 8 (Supplementary Tables S1–S3) and the results corroborated that the time required for the formation of the complex between amylose and iodine was dependent on the PS used. In the presence of **Tri-Py(+)-Me**, **Tetra-Py(+)-Me**, and **Form**, the formation of the dark color (Supplementary Table S1) appeared just after 2–4 min of irradiation, while for **Di-Py(+)-Me *adj*** the iodine-amylose complex was observed after 45 min of irradiation. The formation of the colored complex was not observed for the neutral **Tetra-Py** after 240 min of irradiation and for **Mono-Py(+)-Me** and **Di-Py(+)-Me *opp*** after 75 min of irradiation a slight darkening of the solution was observed.

For the mono-cationic porphyrins **β-ImiPhTPP**, **β-ImiPyTPP**, and **β-BrImiPyTPP** the formation of the deep colored complex was only observed in the presence of **β-ImiPyTPP** after 60 min of irradiation (Supplementary Table S2).

In the assays performed with the non-porphyrinic dyes the combinations **MB**+KI and **RB**+KI promoted the formation of the dark complex after 2–5 min of irradiation and the combination **TBO**+KI after 30 min of irradiation. The combinations of **CV** and **MG** with KI were not able to produce the iodine-amylose complex even after 240 min of irradiation (Supplementary Table S3).

## Discussion

Several studies have shown that aPDT combined with some inorganic salts, namely potassium iodide (Vecchio et al., 2015; Zhang et al., 2015; Huang et al., 2016, 2018a,b,c; Wen et al., 2017) can be potentiated. However, there is not any evidence until now that this potentiation can be observed for all types of PSs, namely cationic porphyrins. In order to gain a more comprehensive knowledge about the potentiation of aPDT by KI, a broad range of PSs were tested in this study.

We started our study by selecting the most effective salt and using as PS the widely studied tetracationic porphyrin 5,10,15,20-tetrakis(1-methylpyridinium-4-yl)porphyrin tetra-iodide (**Tetra-Py^+^-Me**), which is frequently used as standard in aPDT studies. This can be considered a reference for all porphyrinic PSs, since this PS is extensively studied in bacterial photoinactivation processes (Alves et al., 2008; Tavares et al., 2011; Simões et al., 2016). The efficacy of bacterial inactivation by the combination of this PS and the salts KI and NaI was clearly higher than when the PS was used alone, showing that these combinations promoted an increase of the antimicrobial photodynamic efficiency of the PS. On the other hand, no effect was observed with the combinations of **Tetra-Py(+)-Me** with NaBr, KCl, and NaCl during the irradiation time. The loss of efficiency of this porphyrin in these cases could be explained by the fact that bromide and chloride ions retarded the ^1^O_2_ generation, and consequently its action as PS (Keum et al., 2003; Krumova and Cosa, 2016). Therefore, it was obvious that for this PS and under the tested conditions, only salts containing I^−^ as counterion were capable of potentiate the antimicrobial photodynamic inactivation. Similar results were earlier observed when other PSs were tested (Hamblin, 2016). As the combinations PS + KI and PS + NaI were both effective to inactivate the *E. coli*, the potentiation of the others PSs was performed in the presence of the most studied salt KI.

Besides the difficulty of explaining which of the two proposed pathways of decomposition of peroxyiodide produced by the reaction of ^1^O_2_ and I^−^ (see Figure 1) are responsible for the extra microbial killing when KI is present, it was assumed, as proposed previously in other studies, that some information can be taken by the profile of inactivation. If the inactivation curve shows a sharp decrease, free iodine is the main killing species, but if there is a more gradual increase in killing, then there is a contribution from short-lived reactive iodine species (Huang et al., 2018a). Considering the above, we tried to explain the results obtained with the two series of cationic porphyrins, including the neutral **Tetra-Py**, and with the non-porphyrinic PSs. In Table 1 are summarized the results obtained concerning the inactivation profile observed for each combination of KI and PS at 5.0 μM in the photoinactivation of bioluminescent *E. coli*.

**Table 1:** Results obtained in the photoinactivation of bioluminescent *E. coli* using combinations of tested PSs at 5.0 μM and KI.

	Mono-Py(+)-Me	Di-Py(+)-Me *opp*	Di-Py(+)-Me *adj*	Tri-Py(+)-Me	Tetra-Py(+)-Me	FORM	Tetra-Py	β-ImiPhTPP	β-ImiPyTPP	β-BrImiPhTPP	RB	MB	TBO	CV	MG
KI potentiate aPDT?	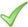	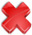	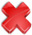	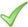	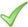	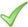	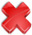	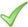	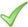	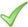	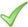	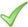	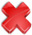	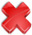	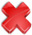
KI causes a sharp decrease in the *E. coli* survival?	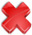	-	-	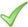	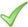	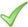	-	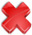	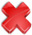	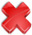	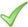	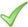	-	-	-

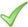
*, Yes;*
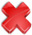
*, No.*

These results allow to classify the PSs studied as: (1) PSs in which its efficiency was potentiated by KI and it was observed a gradual decrease in the *E. coli* survival rate profile [**Mono-Py(+)-Me**, **β-ImiPhTPP**, **β-ImiPyTPP**, and **β-BrImiPyTPP**]; (2) PSs in which its efficiency was potentiated by KI and it was observed an abrupt decrease in the *E. coli* survival rate profile [**Tri-Py(+)-Me,** **Tetra-Py(+)-Me**, **Form**, **RB**, and **MB**]; and (3) PSs in which its efficiency was not potentiated by KI [**Di-Py(+)-Me *opp***, **Di-Py(+)-Me *adj***, **Tetra-Py**, **TBO**, **CV**, and **MG**].

Based on the explanations given in previous works, we can assume that the mechanism of action of the combinations of KI and the PSs **Mono-Py(+)-Me**, **β-ImiPhTPP**, **β-ImiPyTPP**, and **β-BrImiPyTPP** is probably related to the preferential decomposition of the peroxyiodide to the iodine radicals (${\text{I}}_{2}^{\cdot -}$) that, due to their short diffusion distance, cause a gradual decrease in the photoinactivation profile. In the case of **Tri-Py(+)-Me**, **Tetra-Py(+)-Me**, **Form**, **MB** and **RB** the preferential decomposition of the peroxyiodide leads to the formation of free iodine (I_2_/${\text{I}}_{3}^{-}$), which contributes significantly for the abrupt increase observed in the photoinactivation profile of the *E. coli*. This fact was confirmed by the formation of iodine, visible by spectroscopy (Figure 7) and by the color alteration during the irradiation in the presence of starch (Figure 8): PSs that cause a sharp decrease in the *E. coli* survival rate profile revealed higher ability to produce I_2_. On the other hand, the belatedly detection of I_2_ was observed for PSs that cause a gradual decrease in the *E. coli* survival rate profile.

In the cases of PSs in which the efficiency was not potentiated by KI, or was even reduced, we need also to look at other factors that can likewise contribute to this behavior.

The different behavior observed with the dicationic PSs **Di-Py(+)-Me *opp*** (the efficacy was lost in the presence of KI) and **Di-Py(+)-Me *adj*** (no potentiation with KI) (Figures 4B,C) is probably related with their structural features since both isomers have similar capability to generate ^1^O_2_ with high efficiency, as it was described by Simões et al. (2016). Consequently, it can be assumed that both compounds are able to promote the formation of peroxyiodide and its decomposition to iodine radical species (${\text{I}}_{2}^{\cdot -}$). However, for **Di-Py(+)-Me *opp*** these radicals, with a short diffusion distance, probably were not generated close to the target cells and the depletion of ^1^O_2_ by the previous reaction was responsible by losing its previous efficacy. On the other hand, for **Di-Py(+)-Me *adj*** the formation of toxic radicals in close proximity to the target cells can justify the maintenance of its efficacy. However, the toxicity under these conditions was comparable to the previous one in the absence of iodide. The different charge distribution in the two di-cationic porphyrins can explain the different behavior in the presence of KI. A study of Alves et al. (2011a) showed the massive importance of the charge distribution in these two PS efficacies. In this work, the photodynamic inactivation of *E. coli* and *Enterococcus faecalis* using the two isomeric di-cationic porphyrins with different charge distribution showed that the porphyrin with adjacent cationic groups was significantly more active (for both bacteria) than the one with the cationic groups located in opposite *meso* positions. This fact was justified by the distortion of the macrocycle induced by the electrostatic repulsion between the neighboring charged groups in the porphyrin with adjacent cationic groups (Kessel et al., 2003). So, in the case of porphyrinic PSs with cationic groups located in opposite *meso* positions, accompanied by the preferential decomposition of the peroxyiodide to the iodine radicals, as it was observed with **Di-Py(+)-Me *opp***, the addition of KI can even impair the aPDT efficacy. With the porphyrin derivatives **Di-Py(+)-Me *adj***, **Mono-Py(+)-Me**, and **β-ImiPyTPP** the asymmetric distribution of the charge allows the radicals to reach the bacterial cells more effectively. However, the potentiation of the aPDT processes mediated by **Mono-Py(+)-Me** and **β-ImiPyTPP** in the presence of KI but not by **Di-Py(+)-Me *adj*** can also be due to the higher production of free iodine by the two first porphyrins when compared with porphyrin **Di-Py(+)-Me *adj***.

Neutral **Tetra-Py** revealed to be inefficient to photoinactivate *E. coli*, even when KI was used. This can be explained by the fact that this is a neutral PS, and consequently, is not capable to interact with the external membrane of the cell wall of this Gram-negative bacterium. Thus, even when ^1^O_2_ is produced in great amounts, the cytotoxic species will never be close enough to the bacterial cells to cause damage. It is also important to refer that this porphyrin tends to aggregate in aqueous media, making it difficult to act as a PS.

**CV** is known to have an efficient non-radiative deactivation route producing triplet species, such as ^1^O_2_, with low yield and acting mainly through an electron-transfer mechanism (Type I), which causes its bleaching (Docampo et al., 1983; Indig et al., 2000). The results clearly indicate its low efficiency in the photoinactivation of *E. coli*, either when acting alone or combined with KI. These results are justified by its poor ^1^O_2_ production rates allied to its photodegradation when irradiated. Such as in the case of **CV**, it was not surprising that **MG** did not produced any effect in the photoinactivation of bioluminescent *E. coli*, since this PS dye did not produce ^1^O_2_, acting only by the Type I mechanism (Zhuo, 2016). These two PSs dyes show the importance that ^1^O_2_ generation has in the potentiation of aPDT processes mediated by KI. The **TBO** acts mainly by Type II mechanism and, when acting alone inactivate efficiently the bacteria, as **MB** and **RB.** However, when combined with KI, no potentiation was observed. There is, however, a study in the literature reporting the potentiation of the effect of **TBO** by KI, but in this study the **TBO** was tested at 100 μM (Ghaffari et al., 2018). In our case, the concentration of **TBO** was 20 times lower (5.0 μM). These different experimental conditions can justify the differences observed in these two studies. Nevertheless, using NaN_3_ as potentiation agent, the aPDT effect of **TBO** was more effective when compared with the result without the NaN_3_ (Kasimova et al., 2014). **MB** used as the reference for all non-porphyrin dyes, once is the most commonly studied antimicrobial PS in the literature and has received regulatory approval to mediate photodynamic therapy (PDT) of several infectious diseases, acts mainly trough Type II mechanism (Marotti et al., 2010; de Oliveira et al., 2014). Moreover, its aPDT potentiation when combined with KI was already described (Vecchio et al., 2015). Besides that, and according with our results, **MB** can be designated as a PS reference for evaluate the potentiation of these dyes by KI.

It remains unanswered which factor determines whether the mechanism follows *via* formation of iodine radical species (${\text{I}}_{2}^{\cdot -}$) or *via* formation of free iodine (I_2_/${\text{I}}_{3}^{-}$). To answer this question, we cannot neglect other factors that can also contribute for the efficiency of these PSs, such as ^1^O_2_ production, charge number and distribution, aggregation behavior, affinity for the cell membrane.

It is undeniable that the ability of KI to potentiate the aPDT process mediated by some cationic PSs, allows a drastic reduction of the aPDT treatment time as well as the reduction of the PS concentration. However, this potentiation is limited to some PSs and the addition of KI can even impair some PSs. This work helped to elucidate that for the series of compounds studied, the PSs capable to decompose the peroxyiodide into iodine (easily detectable by monitoring the formation of I_2_ through spectroscopy or by the visual appearance of a blue color in the presence of starch) are the promising ones in terms of complementing their efficacy with the action of iodine. Although these studies confirm that the generation of ^1^O_2_ is an important fact in this process, the PS structure, aggregation behavior and affinity for the cell membrane are also important features to take into account.

## Author Contributions

CV performed the antimicrobial photodynamic evaluations assays of all PSs, analysis of biological results and contributed to the manuscript preparation. AG performed the analysis and interpretation of the biological results and contributed to the manuscript preparation. MM and NM performed the synthesis of the porphyrin derivatives. MN and MF were responsible for the supervision of the synthesis of the PSs and contributed in the analysis and interpretation of the biological results and in the manuscript preparation. AA was responsible for the supervision and the design of the antimicrobial photodynamic experiments and contributed in the analysis and interpretation of the biological results and in the manuscript preparation.

## Conflict of Interest Statement

The authors declare that the research was conducted in the absence of any commercial or financial relationships that could be construed as a potential conflict of interest.The reviewer TM and handling Editor declared their shared affiliation at the time of review.
