# Supplementary material for: An Insight Into the Potentiation Effect of Potassium Iodide on aPDT Efficacy
Source: Front Microbiol. 2018 Nov 19;9:2665. doi: 10.3389/fmicb.2018.02665 (PMC6252324; doi:10.3389/fmicb.2018.02665)
Supplement: Supplementary file 1 [file Data_Sheet_1.docx]

**Supporting Information**

An insight into the potentiation effect of potassium iodide on aPDT efficacy

Cátia Vieira^1^, Ana T. P. C. Gomes^1*^, Mariana Q. Mesquita^2^, Nuno M. M. Moura^2^, M. Graça P. M. S. Neves^2^, M. Amparo F. Faustino^2^*, Adelaide Almeida^1^*

^1^ Department of Biology and CESAM, University of Aveiro, 3810-193 Aveiro, Portugal

^2^ Department of Chemistry and QOPNA, University of Aveiro, 3810-193 Aveiro, Portugal

*** Correspondence:**Corresponding Author
[aalmeida@ua.pt](mailto:aalmeida@ua.pt), [faustino@ua.pt](mailto:faustino@ua.pt), [ana.peixoto@ua.pt](mailto:ana.peixoto@ua.pt)


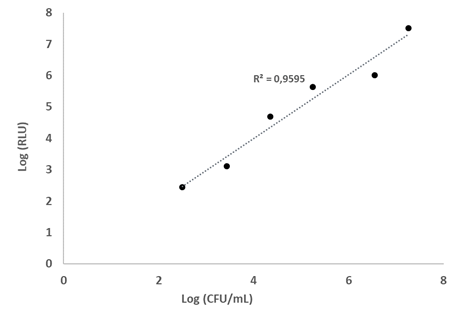


Figure S1. Relationship between the bioluminescence signal and viable counts of an overnight culture of E. coli (<10^9^ CFU/mL) serially diluted in PBS.


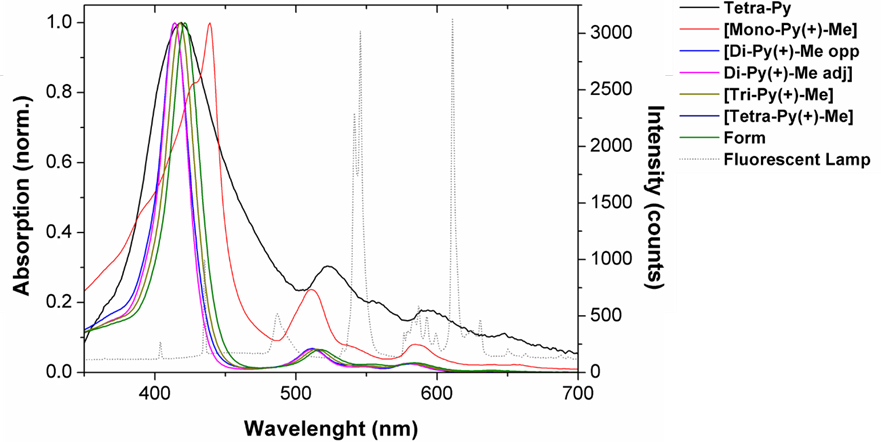


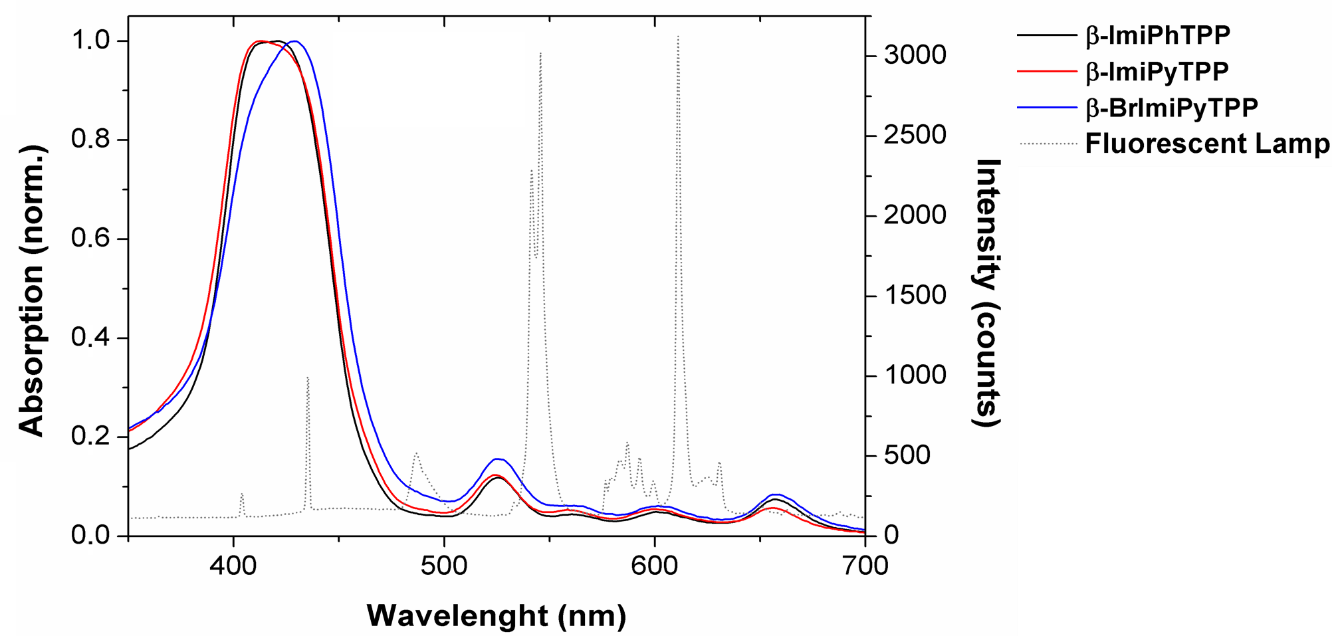


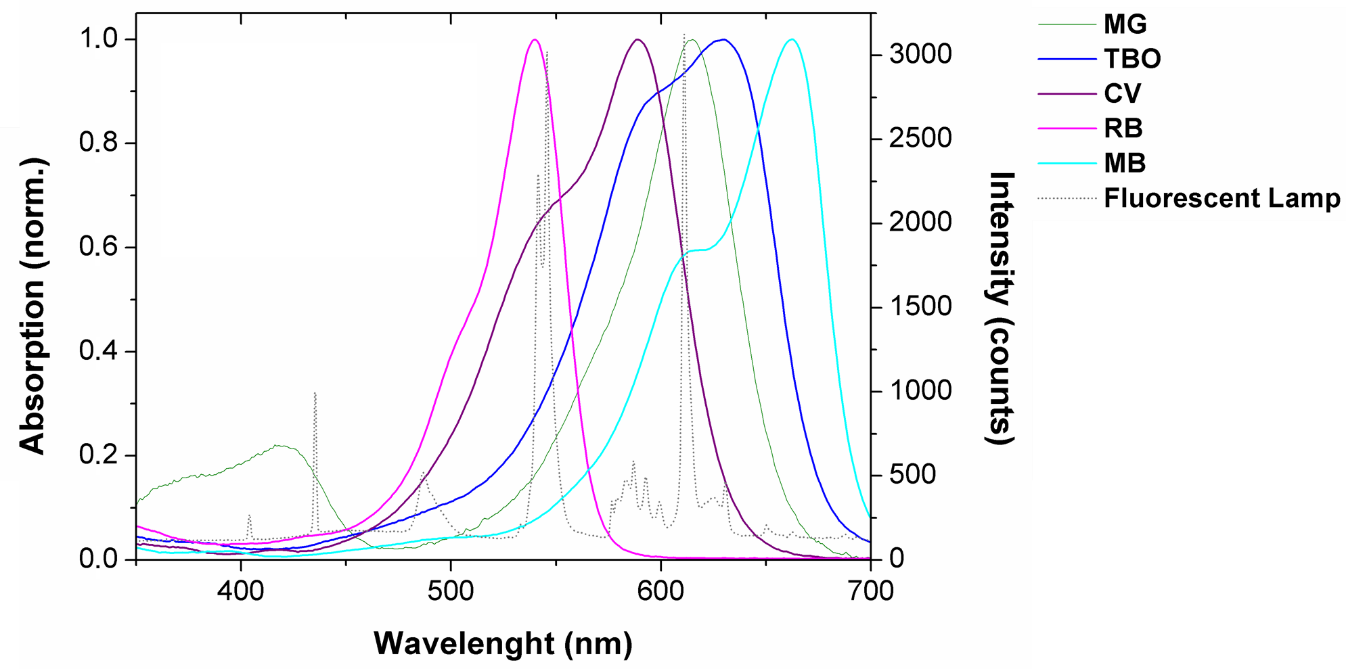


Figure S2. Emission spectrum of light source and absorption spectra of PSs in PBS.

Table S1-: RGB codes corresponding to the colored solutions containing starch solutions after different irradiation periods in the presence of *meso*-substituted porphyrins at 5.0 µM and KI at 100 mM.

| **Irradiation Time (min)** | ***meso*-substituted porphyrins** | | | |
| --- | --- | --- | --- | --- |
|  | **Mono-Py(+)-Me** | **Di-Py(+)-Me adj** | **Di-Py(+)-Me opp** | **Tetra-Py** |
| **0** | 121,122,95 | 124,125,98 | 116,112,80 | 130,132,128 |
| **15** | 122,124,103 | 98,93,70 | 117,114,91 | 131,132,127 |
| **30** | 104,105,94 | 57,55,51 | 101,96,78 | 131,133,127 |
| **45** | 84,84,77 | 36,35,33 | 86,80,65 | 130,132,126 |
| **60** | 66,66,63 | 27,27,27 | 70,65,65 | 130,132,127 |
| **75** | 40,41,48 | 23,23,23 | 56,54,55 | 130,132,127 |
| **90** | 53,54,58 |  | 53,53,53 | 129,132,126 |
| **105** | 52,53,58 |  | 59,56,54 | 129,132,127 |
| **120** | 61,61,63 |  | 64,59,57 | 129,133,127 |

| **Irradiation Time (min)** | ***meso*-substituted porphyrins** | | |
| --- | --- | --- | --- |
|  | **Tri-Py(+)-Me** | **Tetra-Py(+)-Me** | **FORM** |
| **0** | 124,127,106 | 125,124,104 | 111,110,87 |
| **1** | 85,83,74 | 23,22,25 | 115,113,94 |
| **2** | 48,48,49 | 19,18,20 | 109,111,94 |
| **3** | 24,25,26 | 23,23,23 | 72,72,67 |
| **4** | 21,23,23 | 20,20,20 | 39,38,43 |
| **5** | 20,22,21 |  | 32,34,38 |
| **10** | 20,22,20 |  | 28,31,31 |

Table S2-: RGB codes corresponding to the colored solutions containing starch solutions after different irradiation periods in the presence of *beta*-substituted porphyrins at 5.0 µM and KI at 100 mM.

| **Irradiation Time (min)** | ***beta*-substituted porphyrins** | | |
| --- | --- | --- | --- |
|  | **β-ImiPhTPP** | **β-ImiPyTPP** | **β-BrlmiPyTPP** |
| **0** | 126,129,97 | 131,133,107 | 126,129,96 |
| **15** | 94,95,91 | 59,61,57 | 117,118,109 |
| **30** | 94,95,93 | 50,52,49 | 114,115,102 |
| **45** | 76,76,74 | 53,57,55 | 101,102,95 |
| **60** | 80,80,80 | 30,32,33 | 57,61,66 |
| **75** | 51,58,60 | 30,33,33 | 51,58,61 |
| **90** | 40,45,47 | 30,33,33 |  |

Table S3-: RGB codes corresponding to the colored solutions containing starch solutions after different irradiation periods in the presence of non-porphyrinic dyes at 5.0 µM and KI at 100 mM and solution controls

| **Irradiation Time (min)** | **non-porphyrinic dyes** | |  | |
| --- | --- | --- | --- | --- |
|  | **CV** | **MV** | **Positive Control**  (Starch + I_2_) | **Negative Control**  (Starch) |
| **0** | 46,37,101 | 78,133,120 |  |  |
| **240** | 114,113,118 | 72,125,112 | 25,27,26 | 189,189,194 |

| **Time (min)** | **non-porphyrinic dyes** | | |
| --- | --- | --- | --- |
|  | **MB** | **RB** | **TBO** |
| **0** | 71,117,103 | 130,65,96 | 82,112,132 |
| **1** | 48,76,74 | 42,27,42 | 69,99,118 |
| **2** | 33,48,61 | 23,21,27 | 57,101,119 |
| **3** | 26,32,39 | 23,23,23 | 57,98,117 |
| **5** | 23,24,23 | 20,20,20 | 34,57,83 |
| **10** |  |  | 27,39,56 |
| **15** |  |  | 27,36,46 |
| **30** |  |  | 18,19,19 |
